# An economic evaluation of an augmented cognitive behavioural intervention vs. computerized cognitive training for post-stroke depressive symptoms

**DOI:** 10.1186/s12883-015-0522-y

**Published:** 2015-12-29

**Authors:** M. van Eeden, J. A. Kootker, S. M. A. A. Evers, C. M. van Heugten, A. C. H. Geurts, G. A. P. G. van Mastrigt

**Affiliations:** Department of Health Services Research, CAPHRI School for Public Health and Primary Care, Faculty of Health, Medicine and Life Sciences, Maastricht University, P.O. Box 616 6200 MD, Maastricht, The Netherlands; MHeNS, School for Mental Health & Neuroscience; Department of Psychiatry & Neuropsychology, Faculty of Health, Medicine & Life Sciences, Maastricht University, Maastricht, The Netherlands; Donders Centre for Neuroscience, Department of Rehabilitation, Radboud University Medical Centre Nijmegen, Nijmegen, The Netherlands; Department of Neuropsychology & Psychopharmacology, Faculty of Psychology & Neuroscience, Maastricht University, Maastricht, The Netherlands

## Abstract

**Background:**

Stroke survivors encounter emotional problems in the chronic phase after stroke. Post-stroke depressive symptoms have major impact on health-related quality of life (HRQol) and lead to increased hospitalization and therefore substantial healthcare costs. We present a cost-effectiveness and cost-utility evaluation of a cognitive behavioural therapy augmented with occupational and movement therapy to support patients with a stroke with depressive symptoms in goal-setting and goal attainment (augmented CBT) in comparison with a computerized cognitive training program (CogniPlus) as a control intervention.

**Methods:**

A trial-based economic evaluation was conducted from a societal perspective with a time horizon of 12 months. Stroke patients (aged 18+ years) with signs of depression (Hospital Anxiety and Depression Scale (HADS) – subscale depression > 7) were eligible to participate. Primary outcomes were the HADS and Quality Adjusted Life Years (QALYs) based on the three-level five-dimensional EuroQol (EQ-5D-3 L). Missing data were handled through mean imputation (costs) and multiple imputation (HADS and EuroQol), and costs were bootstrapped. Sensitivity analyses were performed to test robustness of baseline assumptions.

**Results:**

Sixty-one patients were included. The average total societal costs were not significantly different between the control group (€9,998.3) and the augmented CBT group (€8,063.7), with a 95 % confidence interval (−5,284, 1,796). The augmented CBT intervention was less costly and less effective from a societal perspective on the HADS, and less costly and slightly more effective in QALYs, in comparison with the control treatment. The cost-effectiveness and cost-utility analyses provided greater effects and fewer costs for the augmented CBT group, and fewer effects and costs for the HADS. Based on a willingness to pay (WTP) level of €40,000 per QALY, the augmented CBT intervention had a 76 % chance of being cost-effective. Sensitivity analyses showed robustness of results.

**Conclusion:**

The stroke-specific augmented CBT intervention did not show convincing cost-effectiveness results. In addition to other literature, this study provided new insights into the potential cost-effectiveness of an adjusted cognitive behavioural therapy intervention. However, as our study showed a 76 % chance of being cost-effective for one outcome measure (QALY) and did not provide convincing cost-effectiveness results on the HADS we recommend further research in a larger population.

## Background

Stroke is a leading cause of death and a source of persistent disability around the world [1]. Annually, 6.7 million people die from stroke, representing 12 % of all global deaths [2]. As a disease of aging, the prevalence of stroke is expected to increase significantly around the world in the years ahead as the global population older than 65 years of age continues to increase by approximately nine million people per year [3]. Stroke survivors often encounter severe cognitive and emotional consequences [4]. Post-stroke depressive symptoms occur frequently in the chronic phase after stroke [5–9]. Recent data from the National Stroke Association (United Kingdom) show that approximately one-third of stroke survivors is affected by varying degrees of post-stroke depression amongst other symptoms [10]. In addition, these symptoms often coincide with increased feelings of anxiety [11]. Besides the major impact on health-related quality of life (HRQol) [12], post-stroke depressive symptoms are associated with increased hospitalization and therefore substantial healthcare costs [13].

Stroke puts a high burden of disease on patients and their caregivers, as well as a considerable financial burden on society. Currently, approximately 3–4 % of total healthcare expenditures in Western countries are spent on stroke [14]. Even greater healthcare expenditures are likely in the near future, with expected increases in the elderly population and the availability of new and better treatments for stroke patients. As healthcare resources are limited and choices have to be made, interest in the economic aspect of stroke and cost-effective stroke-specific interventions has increased in the past few years [15].

Previous research has been used to evaluate different interventions focusing on the treatment of post-stroke depressive symptoms, such as pharmacological interventions [16, 17], yet evidence for the effectiveness of stroke-specific psychological interventions is limited [18]. This is mainly related due to possible lack of efficacy of the interventions under investigation, but also caused by poor study design [19]. There are, however, indications that the use of cognitive behavioural therapy (CBT) might be both effective and cost-effective, based on the treatment of other chronic conditions [20–25]. Psychological interventions, such as CBT, seem promising in terms of effectiveness because they result in fewer side effects than medication and have a stronger effect on preventing relapse of symptoms than pharmacotherapy [20, 26–29]. With respect to psychological interventions, fewer side effects and reduction in relapse are potentially strong indicators for cost-effectiveness. Furthermore, the characteristics of CBT seem to suggest it ought to be an especially good fit to meet the needs of people who suffer from post-stroke depression [17]. Depressed stroke survivors endorse significantly more negative conditions than non-depressed stroke survivors [30, 31] In addition, there is good evidence that remaining active, expressing emotion and finding positive meaning ensures good psychological adjustment in other chronic illnesses [32].

The current study describes the cost-effectiveness and cost-utility of a cognitive behavioural therapy augmented with occupational and movement therapy to support stroke patients with depressive symptoms in goal setting and goal attainment (augmented CBT) for stroke patients suffering from post-stroke depressive symptoms, in comparison with a computerized cognitive training program (CogniPlus) as a control intervention. Our aim was to determine the cost-effectiveness of both interventions from a societal perspective.

## Methods

### Guidelines

The current study and economic evaluation were performed according to the Consolidated Health Economic Evaluation Reporting Guidelines (CHEERS) [33]. The study design and methods were approved by the medical ethical committee of Nijmegen (The Netherlands) and by the executive boards of all participating rehabilitation institutions. The indented time points of assessment were post hoc approved by the same medical ethical committee.

### Design

The current study describes an economic evaluation which was linked to the Restore4stroke CBT study: a multi-centre randomized controlled trial (RCT) conducted in five rehabilitation centres and in the rehabilitation department of one general hospital in the Netherlands. The Medical Ethics Committee of the Radboud University Medical Centre and the executive boards of the participating institutes approved the study. The inclusion of participants took place between February 2012 and October 2013. All patients provided written informed consent. The RCT was registered in the Dutch Trial Register as NTR2999. Detailed information on the methods of this RCT and the current study can be found elsewhere [15, 34].

### Eligibility criteria

Patients where included if they: (1) suffered any type of stroke in the last 3 months and complaints in mood (indicating possible symptoms of anxiety or depression) occurred post stroke, (2) were 18 years or older, (3) scored >7 on the HADS depression subscale (HADS-D) [35], (4) mastered the Dutch language, and (5) had sufficient communication skills based on the Mini-Mental State Examinate [36] > 27 and had normal score (>0) on communication items of the National Institutes of Health Stroke Scale items [37]. Patients were excluded if they: (1) scored < 19/20 on the Barthel Index [5] indicating premorbid disabilities, (2) were staying in an in-patient setting, (3) suffered from co-morbidity that might affect outcomes (e.g. cancer or major psychiatric illnesses for which psychological treatment was being given at the moment of inclusion), (4) were diagnosed with a major depression requiring medication, or (5) were diagnosed with a pre-morbid depression or had received psychiatric care for depression.

### Interventions

The augmented CBT intervention was individually administered and tailored according to specific subjects-own activity-related goals. These goals could capture domains as e.g. self-care, leisure, household, work, and improvement of mobility-related goals such as movement and walking. The CBT treatment program consisted of 10–12 sessions with a certified healthcare psychologist, experienced in the treatment of depression and stroke rehabilitation. The augmentation part of the intervention was specifically aimed at supporting patients in goal setting and goal attainment. This part consisted of three sessions of occupational therapy (OT) or movement therapy (MT) provided by an occupational or movement therapist. Each of these sessions was comprised of 20–25 min blocks divided by a 10–15 min break. If a patient’s score on the HADS subscale anxiety (HADS-A) was > 7, the protocol was expanded with an additional (fourth) OT/MT session [38]. All treatments were provided at the closest participating centre and the complete program was executed within 4 months.

The control group was provided with an individual, patient-tailored computerized cognitive training program (CogniPlus) [39]. In specific self-determined cognitive domains (e.g. attention, memory, executive functioning, and visual attention) patients executed computer tasks on their own performance level. This impairment-oriented intervention consisted of 13–16 sessions provided at participating centres under the supervision of a research assistant or psychological assistant, for a period of 4 months. We chose to compare the augmented CBT intervention to an ‘active’ intervention (and not usual care) to control for Hawthorne effects. Evidence from Spikman et al. [40] showed that a similar control group did not improve in executive functioning, and that generalisation of what was assessed in the control intervention to daily life did not occur. Further information on the justification of both interventions can be found elsewhere [15, 34].

### Procedure

Treating physicians and healthcare psychologists of the participating institutes were informed about the inclusion and exclusion criteria of the study. Accordingly, they recruited eligible patients at participating centres. Potential participants were contacted by the primary researcher (JK) and informed about the intervention. In case of a positive reply, an individual appointment was made to confirm the inclusion and exclusion criteria, to obtain written consent, and to conduct the baseline assessment. Patients were randomly allocated to either the augmented CBT group or control group. Stratified block randomization (block size four) was conducted, in which level of anxiety was a minimization factor (HADS-A > 7 vs ≤ 7). Follow-up assessments took place at the nearest participating centre for each patient and were conducted by a research assistant who was blinded to the type of intervention provided to the patient. Prior to each assessment, the assessor explicitly asked patients not to talk about the content of their intervention. The success of assessor blinding was ensured with a short self-constructed questionnaire at the end of each assessment.

### Time horizon

Patients were randomly allocated to one of the interventions after the baseline assessment (T0). Assessments took place post treatment (T1), at 8 months follow-up (T2) and at 12 months follow-up (T3).

### Sample size

Based on a minimal detectable effect size of 0.6 the standard deviation (SD) on the HADS (α = 0.05; β = 0.80), a minimum of 45 participants per group was required. With an expected dropout of 15 %, 106 participants needed to be recruited. However, due to recruitment difficulties we were not able to include the minimum amount of participants necessary. Reconsidering the proposed analysis of the Restore4stroke CBT study, it was decided to add the T1 measurement next to T2 to estimate the effect of treatment outcomes. These repeated measures at T1 and T2 would reduce variance, double the power and reduce the required sample size. Therefore, a new power (*n* = 53; α = 0.05; β = 0.80) allowed us to continue with fewer participants than originally planned. The Medical Ethics Committee approved an amendment containing these changes.

### Outcome measures

The main outcome for the cost-effectiveness analysis (CEA) was depression and anxiety, assessed by the HADS (total score). The HADS is a 14-item questionnaire (seven questions concerning ‘depression’ and seven questions concerning ‘anxiety’) on a 4-point scale. Higher scores on the HADS indicate greater levels of depression and/or anxiety. The validity and reliability of the HADS has been determined in previous research [41].

The main outcome for the cost-utility analysis (CUA) was health-related quality of life (further referred to as QoL), as measured by with the five-dimensional three-level EuroQol (EQ-5D-3 L) [42]. The five EuroQol dimensions are mobility, self-care, usual activities, pain/discomfort and anxiety/depression. To estimate the utility of health states described by patients, we used the Dutch tariff [43]. Quality adjusted life years (QALYs) were calculated by means of the area under the curve method. Higher QALYs indicate more improvement in QoL.

### Resource use and costs

A 19-item self-report cost-questionnaire was constructed to collect cost data from a societal perspective, based on the steps described by Thorn [44]. The validity and feasibility of generic self-report questionnaires has been investigated elsewhere [45]. As a societal perspective is a broad perspective in which all relevant costs for the whole population are incorporated [46], we included four main cost categories in this study: intervention, healthcare, patient- and family-related, and productivity costs. A bottom-up approach was used to determine intervention costs (e.g. intervention materials, consultation hours with a psychologist). Healthcare costs covered care provider utilization (e.g. general practitioner and medical specialist consultations), complementary medicine, home care and medication. As a guideline for calculating healthcare costs we used the Dutch Manual for Costing [47].

Medical and personal aids were based on costs per user within the aid category provided by the Dutch care institute [http://www.gipdatabank.nl] and the costs of prescription medicines were valued by the price per dosage for drug costs in the Netherlands [48, 49]. Travel costs and costs of informal care were included as patient- and family costs. Travel costs were calculated by multiplying the average distance with standard price weights provided by The Dutch Manual for Costing [47], corrected for the costs of public transport and parking costs. Shadow prices were used to determine the costs of informal care, which were equal to the hourly wage rates of professional caregivers (i.e. housekeepers). Productivity costs were valued according to the human capital approach. This approach states that productivity costs are calculated by multiplying the number of sick days by the costs of labour, corrected for different age categories. The human capital approach is the international standard in calculating productivity costs, whereas its counterpart, the friction cost method, is subject to variability in the national economic cycles [50, 51]. Furthermore, due to changes in Dutch legislations, it is unlikely for employees that they are being replaced, making it imperative to include long-term absenteeism as well.

### Currency, price date and conversion

All costs reported in this study were expressed in Euro’s (€). Consumer price indices were used to adjust all costs to the index year 2012 [52]. Discounting was not necessary since the follow-up period of the current study did not exceed 1 year.

### Analytic methods

Intention-to-treat was used to analyse data. Missing values for resource use were handled by individual mean imputation, the recommended imputation methods on dealing with intermittent data in economic evaluation studies [53]. Missing data on the main outcomes were handled by multiple imputation (MI). MI is a technique often used to analyse data sets with missing values; it is the process of replacing each missing data point with a set of plausible values to generate complete data sets [54]. Since baseline utility measurements are included when calculating QALYs we consider any baseline difference in utility scores as a potential bias, regardless of whether or not this difference is significant or not. Therefore, we used a regression based correction method to correct for baseline differences in utility scores [55].

An incremental cost-effectiveness ratio (ICER) was calculated by dividing the incremental costs by the incremental effects, and an incremental cost-utility ratio was calculated by dividing the incremental costs by the differences in QALY. We used non-parametric bootstrapping (5000 replications) to estimate the uncertainty surrounding the ICER, due to the highly skewed cost distribution. Bootstrapped cost-effectiveness and cost-utility pairs were presented in cost-effectiveness planes (CE-planes). Statistically significant differences in costs were determined by means of a 95 % confidence interval (further referred to as CI). If the CI entailed a ‘0’ value, no statistical differences in costs were found.

Furthermore, a cost-effectiveness acceptability curve (CEAC) was made to express the probability of the augmented CBT intervention being a cost-effective alternative in comparison with the control intervention. A CEAC shows the probability of an intervention to be a cost-effective alternative for a certain threshold; the amount of money society is willing to pay (WTP) to gain one unit of effect (e.g. a one-point improvement on the HADS or one QALY). For the HADS, the WTP threshold is an unknown quantity. A previous study on manual psychological therapy for dementia patients used a WTP level of €500 per one-point improvement on the HADS [56]. The minimal important difference of the HADS has not been established, but in patients with chronic obstructive pulmonary disease (COPD) a minimal important difference of 1.6 was found [57]. The WTP threshold for a QALY differs per country or even within countries. In the Netherlands, the Dutch Council of Public Health and Care published a report in 2006 regarding the burden of disease in the Netherlands, estimating a QALY threshold for stroke at €40,000 Euros [58]. All statistical analyses were performed using the Statistical Package for the Social Sciences (SPSS) version 21 or Microsoft Excel (bootstrapping).

### Sensitivity analyses

To test the robustness of assumption made in the base case analyses, we conducted four one-way sensitivity analyses. First, the price for a rehabilitation day treatment was decreased to €117, equal to a regular rehabilitation consultation. Second, the friction cost method was used to calculate productivity costs instead of the human capital approach [47]. Third, we compared our base case societal perspective for calculating costs with a healthcare perspective. Finally, as different sets of tariffs exist to calculate utilities, we analysed the impact of the use of Dutch tariffs versus United Kingdom (UK) tariffs [59].

## Results

### Sample

**Fig. 1:**
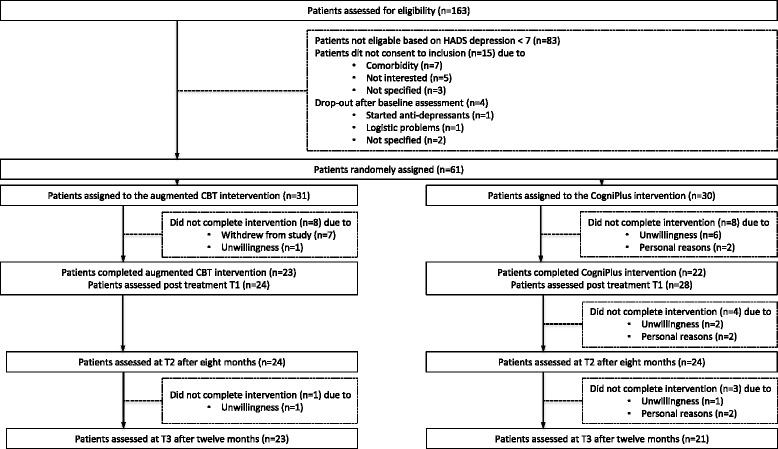
Inclusion of patients

One hundred sixty-three patients were assessed regarding their eligibility for participation in this study (Fig. 1). Eighty-three patients were found ineligible based on their HADS-D score (≤7) and 15 patients did not meet other inclusion criteria. After baseline measurement, four patients dropped out due to various reasons. Thus, a total of 61 patients were included in this study (Table 1). Thirty-one patients (52 %) were allocated to the augmented CBT group and 30 patients (48 %) to the control group. As presented in Fig. 1, the percentage of missing data at T1 was 15 % (*n* = 9), at T2 was 21 % (*n* = 13) and at T3 was 28 % (*n* = 17).

**Table 1:** Baseline patients’ characteristics (*n* = 61)

	Augmented CBT (*n* = 31)		CogniPlus (*n* = 30)	
*Demographic characteristics*	n.		n.	
Mean age in years (SD^1^)	31	62.2 (8.3)	30	60.0 (10.5)
Gender (% men)	31	61.3	30	63.3
Paid work (%)	31	29	30	43.3
Hours worked/week (SD)	31	2.3 (5.6)	30	5.5 (12.8)
*Stroke characteristics*				
Time since stroke in months (SD)	31	43.9 (51.1)	30	40.2 (41.9)
Type of stroke (% infarction)	28	75	28	85.7
Affected hemisphere (% right)	28	30.8	26	39.3
*Outcome measures*				
Effects				
HADS Total (SD)	31	22.3 (6.2)	30	22.3 (6.7)
HADS Anxiety (SD)	31	9.9 (4.1)	30	10.0 (4.4)
HADS Depression (SD)	31	12.5 (3.3)	30	12.33 (3.4)
Utility (SD)	31	0.58 (0.27)	30	0.47 (0.30)
Costs				
Healthcare costs, € (SD)	31	2,213.4 (880.9)	30	2,051.8 (501.6)
Non-healthcare costs, € (SD)	31	2,528.3 (712.4)	30	1,962.3 (518.1)
Total societal costs, € (SD)	31	4,717.7 (1,203.9)	30	4,038.4 (853.7)

^1^SD: standard deviation

### Cost analysis

The average total societal costs were greater for the control group (€9,998) in comparison with the augmented CBT group (€8,064), but this difference between the two groups was not significant (CI: −5284, 1,796). Healthcare costs were also greater for the control group (€5,055 compared to €3,771), but this difference was not significant either (CI: −3,039, 465). Specialist visits was the only cost category within healthcare costs that showed significantly greater costs for the control group (€993) in comparison with the augmented CBT group (€539) (CI: −868, −38). The non-healthcare costs were also in favour of the intervention group (€4,926 in comparison with €4,333 in the control group), however, no significant difference was found (CI: −2,778, 1,551). Both the productivity costs (CI: −3,065, −93) and the productivity costs of the caregiver (CI: −1,354, −27) were significantly greater for the control group. As expected, intervention costs were greater for the augmented CBT group (€1,130 in comparison with €592). Further details are presented in Table 2.

**Table 2:** Average resource use and costs per category over 12 months (bootstrapped)

		Augmented CBT intervention (*n* = 31)		CogniPlus intervention (*n* = 30)		
Category	Unit price	Average use (SD)	Average costs, Euros (SD)	Average use (SD)	Average costs, Euros (SD)	95 % CI^1^
*Healthcare*						
Hospital	Night	0.5 (2.3)	255.9 (187.7)	1.6 (4.2)	787.8 (370.9)	(−1,428, 173)
Rehabilitation centre	Night	0.0 (0.0)	0.0 (0.0)	0.0 (0.0)	0.0 (0.0)	(0,0)
Nursing home	Night	0.0 (0.0)	0.0 (0.0)	0.0 (0.0)	0.0 (0.0)	(0,0)
General practitioner	Consultation	9.6 (9.2)	285.8 (48.2)	10.8 (10.6)	328.2 (55.6)	(−186, 93)
Specialist	Consultation	4.2 (5.1)	538.8 (113.1)	7.8 (7.9)	993.1 (172.2)	(−868, −38)^2^
Physiotherapy	Consultation	23.5 (34.0)	893.2 (233.3)	26.5 (31.7)	1,006.1 (209.8)	(−702, 525)
Remedial therapy	Consultation	5.5 (11.6)	207.0 (79.8)	4.8 (9.6)	177.3 (62.6)	(−166, 234)
Mensendieck	Consultation	0.5 (2.9)	20.4 (19.1)	0.0 (0.0)	0.0 (0.0)	(0, 58)
Occupational therapy	Consultation	2.8 (5.4)	64.1 (22.1)	0.9 (2.9)	20.4 (12.3)	(−2, 98)
Activity therapy	Consultation	1.3 (5.4)	46.1 (35.2)	4.2 (13.6)	155.4 (92.6)	(−330, 57)
Speech therapy	Consultation	2.6 (10.9)	88.5 (65.7)	1.4 (5.6)	50.2 (35.7)	(−88, 209)
Social work	Consultation	0.2 (0.8)	12.9 (9.7)	1.0 (3.3)	66.4 (39.9)	(−140, 9)
Psychologist	Consultation	7.0 (8.6)	581.6 (126.4)	5.9 (7.7)	487.7 (115.7)	(−231, 430)
Psychiatric nurse	Consultation	0.0 (0.0)	0.0 (0.0)	0.2 (0.9)	6.4 (5.0)	(−18, 0)
Psychiatrist	Consultation	0.0 (0.0)	0.0 (0.0)	0.2 (0.9)	17.8 (18.0)	(−55, 0)
Rehabilitation day treatment	Day	1.4 (5.5)	375.9 (264.2)	2.3 (6.9)	591.2 (317.2)	(−1,034, 542)
Medication	Other		410.0 (114.2)		374.3 (108.5)	(−234, 208)
Subtotal			3,771.3 (551.5)		5,055.3 (713.0)	(−3,039, 465)
*Non-healthcare*						
Travel costs	Other		289.4 (48.9)		301.3 (41.2)	(−137, 119)
Productivity costs	Hours/week	0.3 (1.2)	243.5 (206.4)	1.7 (4.3)	1,648.8 (728.5)	(−3,065, −93)^2^
Productivity costs caregiver	Hours/week	0.0 (0.1)	12.9 (12.4)	0.6 (2.1)	615.2 (347.8)	(−1,354, −27)^2^
Paid help	Hours	45.9 (71.2)	1,728.8 (477.2)	28.2 (53.3)	1,061.2 (368.9)	(−465, 1,790)
Unpaid help	Hours	60.4 (141.9)	813.7 (315.7)	40.3 (86.7)	539.9 (204.7)	(−483, 261)
Tools^3^	Item		157.0 (54.0)		78.6 (33.5)	(−46, 209)
Home adjustments^3^	Item		0.0 (0.0)		134.5 (120.2)	(−400, 0)
Subtotal			4,333.6 (745.7)		4,926.0 (835.3)	(−2,778, 1,551)
*Intervention costs*	Other		1,129.8		592.1	
Total societal costs			8,063.7 (1,126.1)		9,998.3 (1,370.1)	(−5,284, 1,796)

^1^significant difference

^2^95 % Confidence Interval level

^3^Tools: e.g. brace, special glasses; Home adjustments: e.g. toilet or shower adjustment

### Cost-effectiveness and cost-utility

**Fig. 2:**
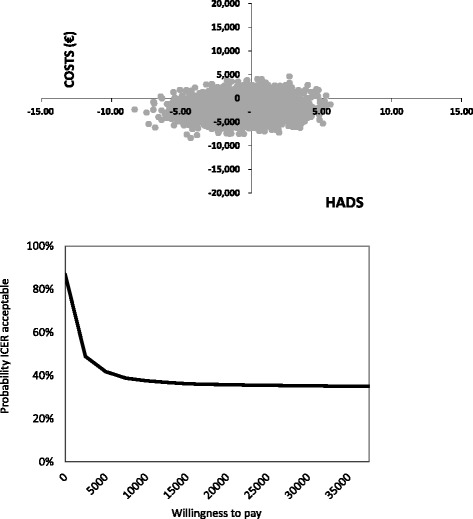
Cost-effectiveness plane HADS and cost-effectiveness acceptability curve HADS

The CEA for the HADS showed that the augmented CBT group induced fewer costs (−€1,913) but also fewer effects (−0.8), resulting in an ICER of €2,395 (Table 3). The majority of bootstrapped ICERs (58 %) were located in the southwest (SW) quadrant of the CE-plane (Fig. 2) indicating fewer costs and fewer effects for the augmented CBT intervention, and 29 % of the bootstrapped ICERs were located in the dominant southeast (SE) quadrant indicating greater effects and fewer costs for the augmented CBT intervention.

**Table 3:** Mean cost and effect differences between the Augmented CBT group and CogniPlus group, incremental cost-effectiveness ratios and cost-effectiveness plane distributions

		Sample size		ΔCosts	ΔEffects	ICER^1^	Distribution cost-effectiveness plane (quadrant, %)^2^			
Analysis	Effect measure	Augmented CBT	CogniPlus	Euro			NE	SE (dominant)	SW	NW (inferior)
Cost-effectiveness	HADS	31	30	−1,912.6	−0.8	2,395.3	5	29	58	8
Cost-effectiveness	QALY	31	30	−1,912.6	0.01	−160,389.9	5	55	31	9

^1^ICER: incremental cost-effectiveness ratio

^2^NE (northeast quadrant): SM more effective and more costly compared to EDU

SE (southeast quadrant): SM more effective and less costly compared to EDU

SW (southwest quadrant): SM less effective and less costly compared to EDU

NW (northwest quadrant): SM less effective and more costly compared to EDU

**Fig. 3:**
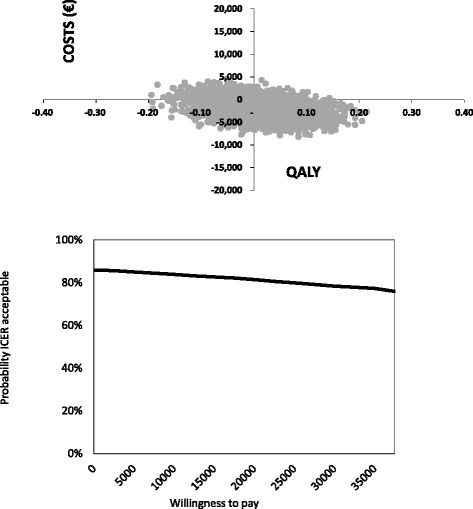
Cost-effectiveness plane QALY and cost-effectiveness acceptability curve QALY

Patients in the augmented CBT group gained slightly more QALYs (mean: 0.01) compared to control group patients. More QALYs gained combined with fewer societal costs (−€1,913) induced by the augmented CBT group resulted in a dominant ICER. As presented in Fig. 3, 31 % of the bootstrapped ICERs were located in the SW quadrant (fewer costs and effects) of the CE-plane and 55 % of the bootstrapped ICERs were located in the dominant SE quadrant (fewer costs and greater effects).

The cost-effectiveness acceptability curves (CEACs) of the HADS and QALY are also presented in Figs. 2 and 3. The slope of the HADS CEAC indicates that with a WTP threshold of €2,500, the probability of the augmented CBT intervention being cost-effective was 49 %. Furthermore, greater WTP levels showed an increasing decline in the probability of the augmented CBT intervention being cost-effective. In the Netherlands, there is no fixed WTP threshold for a QALY, but the threshold is dependent on the burden of disease [58]. Based on the burden of stroke, a WTP level of €40,000 was considered acceptable [58]. Using this threshold, the QALY CEAC indicates that there is a 76 % chance that the augmented CBT intervention will be cost-effective.

### Sensitivity analysis

A sensitivity analysis was performed to estimate the impact of reducing the price of a rehabilitation treatment day, on the cost-effectiveness results (Table 4). The analysis proved robustness of the base case assumption for this parameter for both the HADS and QALY, as it resulted in a slightly lower ICER and no major changes in distributions on the CE-plane (7. 4). Calculating productivity costs by means of the friction cost method resulted in a much lower ICER for both the HADS and QALY and major shifts in distributions on the CE-plane to both ‘west’ quadrants (indicating fewer costs for the augmented CBT group). Estimating total costs from a healthcare perspective resulted in lower ICERs for both outcome measures; this was due to fewer cost categories being included. A major shift on the CE-plane of the QALY is noticeable towards the northeast (NE) quadrant (greater costs and greater effects). Finally, UK tariffs were used to calculate QALYs resulting in a decrease in ICER, and again, a shift on the CE-plane towards the SE quadrant.

**Table 4:** Sensitivity analysis

Analysis^1^	ΔCosts (€)	ΔEffects	ICER^2^	Distribution cost-effectiveness plane (quadrant, %)^3^			
				NE	SE (dominant)	SW	NW (inferior)
Base case HADS	−1,912.6	−0.8	2,395.3	5	29	58	8
Unit price day of rehabilitation	−1,787.0	−0.8	2,238.0	6	29	57	8
Friction costs	−796.4	−0.8	997.4	12	23	46	19
Healthcare perspective	−1,281.4	−0.8	1,604.7	3	31	61	5
Base case QALY	−1,912.6	0.01	−160,389.9	5	55	31	9
Unit price day of rehabilitation	−1,787.0	0.01	−149,859.6	4	54	33	9
Friction costs	−796.4	0.01	−66,784.4	12	46	24	18
Healthcare perspective	1,281.4	0.01	107.454.7	52	6	6	36
QALY UK tariff	−1,912.6	0.04	−51.797.4	7	65	22	6

^1^Base case analysis values a day of rehabilitation day as a hospital treatment day (€266.53), calculates production costs by means of human capital method, uses the societal perspective to calculate total costs

corrects for baseline differences with regression analysis and calculates utilities with a Dutch tariff. Sensitivity analyses values a rehabilitation treatment day as a rehabilitation contact (€116.81), calculates production

costs with the friction cost method, estimates total cost from a healthcare perspective calculates utilities with a UK tariff

^2^ICER: incremental cost-effectiveness ratio

^3^NE (northeast quadrant): SM more effective and more costly compared to Edu

SE (southeast quadrant): SM more effective and less costly compared to Edu

SW (southwest quadrant): SM less effective and less costly compared to Edu

NW (northwest quadrant): SM less effective and more costly compared to Edu

## Conclusions

To our knowledge, this is the first cost-effectiveness analysis performed on a prospective randomized comparison of an augmented CBT intervention versus computerized cognitive training (CogniPlus) for post-stroke depressive symptoms.

The results of this study provides evidence that, using the HADS as an outcome, the augmented CBT intervention for stroke patients was less costly and less effective from a societal perspective and less costly and slightly more effective in terms of QALYs, compared to the control treatment. Cost differences between the two groups could be explained by costs of admission to a hospital, specialist consultations and home adjustments, but the larger part of the difference in total costs was due to productivity costs of both the patient and the caregiver which were both significantly less in the augmented CBT group. The fact that, at baseline, 43.3 % of the control group had paid work, in comparison with only 29 % of the augmented CBT group, and that patients in the control group worked more than twice the number of hours per week might explain this difference.

The results of the cost-effectiveness analysis showed that the probability of the augmented CBT intervention being cost effective was 49 % for a WTP of €2,500. No fixed WTP threshold for the HADS exists, but previous research used a WTP of €500 per point improvement on the HADS based on expert opinion [56]. A dominant ICER for the QALY was found, indicating greater effects and fewer costs for the augmented CBT group. Combined with a high probability of the augmented CBT intervention being cost-effective, the results from the cost-utility analysis were in favour of the augmented CBT intervention. However, due to the minimal difference in effects (mean: 0.1), these results should be interpreted with caution.

In general, the sensitivity analyses showed robustness of results. For the HADS, a distinct shift in the distribution of bootstrapped ICERs was noticeable when calculating productivity costs with the friction method. This might be expected, since the friction cost method accounts for a shorter period of productivity losses resulting in fewer costs, and a lower ICER with the same difference in HADS score and a slightly greater probability of the augmented CBT intervention being cost-effective. This was also observed for the friction method, employed as a sensitivity analysis with regard to QALYs. A major shift was noticeable when conducting the cost-utility analysis from a healthcare perspective instead of the societal perspective. It appeared that the augmented CBT group induced both greater costs and greater effects leading to a shift on the CE plane towards the NE quadrant. This, and the fact that the difference in effect was minimal, indicated that the majority of total costs for the control group were accounted for by non-healthcare costs.

To our knowledge, this current study provides new but preliminary evidence on the cost-effectiveness of a stroke-specific augmented cognitive behavioural therapy. Four previous studies investigating a comparable intervention in different populations showed similar results [22–24, 27]. More precisely, recent research on the effectiveness of an online CBT intervention for depressive patients found greater costs for CBT group and a cost per QALY gained of €23.857, which is below the acceptable UK threshold of €27.784 per QALY [21]. Although this study was conducted from a National Health Services (NHS) perspective and the CBT intervention was compared with care as usual, the conclusions drawn from this study where in line with the current study results, indicating the potential cost-effectiveness of CBT. Another study evaluating the effectiveness of CBT for depressive patients in a non-stroke population reported greater costs and greater effects in terms of QALYs, resulting in a base case ICER of €20.714 [25]. Two other studies on the cost-effectiveness of CBT for patients with low-back pain and for people with schizophrenia-spectrum disorder also showed the possible cost-effectiveness of cognitive behavioural therapy [22, 24]. It is important to note that these previous studies chose ‘care as usual’ as comparator, whereas the current study chose a computerized training (CogniPlus). The choice of comparator is a critical design parameter in economic evaluation research and may influence study results [60].

### Strengths and limitations

To our knowledge, there has been no previous economic evaluation research on a stroke-specific cognitive behavioural therapy. Furthermore, the cost-effectiveness and cost-utility analyses in this study were conducted from a societal perspective, which is also considered to be a strength because it provides extensive evidence on a broad range of costs. Finally, both the outcome assessors and research assistants were blinded for the randomised treatment.

The current study was also subject to several limitations. First, due to recruitment difficulties we were not able to include the desired amount of participants, which was the reason for performing a new power calculation. Still, the current study results must be interpreted with caution due to the small sample size. Next, we had to deal with a considerable number (*n* = 17; 28 %) of missing values on T3 measurement concerning the HADS and EQ-5D-3 L. A multiple imputation (MI) method was used to handle these missing values [54]. There are alternative methods for imputation, such as linear mixed models, but since cost-effectiveness analysis requires individual patient data we believe that MI was the best method for the analyses performed in the current study. Also, productivity costs of the caregivers were estimated with a mean hourly wage and a mean age, since information with respect to the employment of caregivers was limited. Finally, we found and measured differences between interventions within the time horizon chosen for this study. However, with progressing insight, we would argue that it would be interesting to expand the time horizon, by adding extra follow-up measurement moments or using modelling techniques in further research in order to identify the long-term effects of both interventions in comparison to each other.

### Concluding remarks

Taking into account the limitations of the current study, we conclude that the preliminary results of the cost-effectiveness of the stroke-specific augmented CBT intervention under investigation were not convincing. As our study showed a 76 % chance of being cost-effective for one outcome measure (QALY) and did not provide convincing cost-effectiveness results on the HADS. However, other studies showed the potential for the augmented CBT intervention to be cost-effective in treating depression. Although we have argued why we have chosen an ‘active’ control intervention, it would be very interesting to investigate the effect of including a care as usual group as third study arm in future research. Also, in addition to other literature, this study provided new insights into the potential cost-effectiveness of an adjusted cognitive behavioural therapy intervention. Therefore, for further research we would recommend recruitment of a larger stroke population, i.e. in multiple sites.

## Abbreviations

HRQol: Health-related quality of life
CBT: Cognitive behavioural therapy
HADS: Hospital anxiety and depression subscale
QALY: Quality-adjusted life year
EQ-5D-3 L: Three-level five-dimensional EuroQol
UK: United Kingdom
ICER: Incremental cost-effectiveness ratio
WTP: Willingness to pay
CHEERS: Consolidated health economic evaluation reporting standards
RCT: Randomized controlled trial
MMSE: Mini-mental state examinate
NIHSS: National Institute of Health Stroke Scale
OT: Occupational therapy
MT: Movement therapy
SD: Standard deviation
JK: Joyce Kootker
CEA: Cost-effectiveness analysis
CUA: Cost-utility analysis
GP: General practitioner
MI: Multiple imputation
CE-plane: Cost-effectiveness plane
CI: Confidence interval
CEAC: Cost-effectiveness acceptability curve
SPSS: Statistical Package for the Social Sciences
NW: Northwest
SW: Southwest
SE: Southeast
NE: Northeast
NHS: National Health Services
